# Association between orthodontic treatment and the occurrence of temporomandibular disorders: A systematic review and meta-analysis

**DOI:** 10.4317/jced.59970

**Published:** 2022-12-01

**Authors:** Franz-Tito Coronel-Zubiate, Consuelo Marroquín-Soto, Luis-Alberto Geraldo-Campos, Rubén Aguirre-Ipenza, Laura-Madeleine Urbano-Rosales, Sara-Antonieta Luján-Valencia, José-Giancarlo Tozo-Burgos, Heber-Isac Arbildo-Vega

**Affiliations:** 1 Faculty of Health Sciences, Universidad Nacional Toribio Rodríguez de Mendoza de Amazonas. Chachapoyas, Peru; 2 School of Stomatology, Universidad Científica del Sur. Lima, Peru; 3 Research Directorate, Universidad Privada Peruano Alemana. Lima, Perú; 4 Faculty of Health Sciences, Universidad Continental. Lima, Peru; 5 Postgraduate School, Universidad Peruana Cayetano Heredia. Lima, Peru; 6 Faculty of Dentistry, Universidad San Martín de Porres. Arequipa, Perú; 7 Biofunctional Aesthetic Dentistry Center. Tacna, Peru; 8 Faculty of Dentistry, Department of General Dentistry, Universidad San Martín de Porres. Chiclayo, Perú; 9 Faculty of Human Medicine, Department of Human Medicine, Universidad San Martín de Porres. Chiclayo, Perú

## Abstract

**Background:**

Temporomandibular disorders (TMD) are related to a series of clinical manifestations that appear in the form of pain. Currently, there is controversy about the appearance of TMDs after orthodontic treatment. Therefore, the objective of the present study was to determine the association between orthodontic treatment and the occurrence of Temporomandibular Disorders (TMD).

**Material and Methods:**

A bibliographic search was carried out until April 2022, in the following electronic databases: Pubmed/Medline, Scopus, Scielo, Google Scholar and Web of Science. We included studies that were case-control and cohort studies, dealing with the association between orthodontic treatment and TMD, in English and Spanish, and with no time limit. The Newcastle-Ottawa scale was used to assess risk in the included studies. In addition, RevMan 5.3 was considered for meta-analysis, using as a measure the ODDS ratio in a random-effects model with a 95% confidence interval.

**Results:**

The preliminary search yielded a total of 686 articles, discarding those that did not meet the selection criteria, leaving only 6 articles. These studies reported that there is a significant association between orthodontic treatment and the occurrence of TMD, equivalent to an ODDS ratio of 1.84 with a confidence interval of 1.19-2.83.

**Conclusions:**

It is concluded that there is an association between orthodontic treatment and the occurrence of TMD, therefore, a person undergoing orthodontic treatment is up to 1.84 times more likely to develop TMD.

** Key words:**Orthodontic treatment, temporomandibular disorders, orthodontics, cases and controls, cohorts, review, Meta-Analysis.

## Introduction

Temporomandibular Disorders (TMD) are related to a number of clinical manifestations that present in the form of pain as well as dysfunction of the temporomandibular joints (TMJ) and masticatory muscles (1). Based on the Diagnostic Criteria for Temporomandibular Disorders (DC/TMD), 12 different conditions are established for temporomandibular disorders (TMD), which are: muscle pain, local muscle pain, facial muscle pain with reduction, joint pain, TMD-related headache, disc pain with reduction, disc pain with intermittent blockage, disc pain without reduction (2). Typical TMD patients are women of childbearing age (3). The increased incidence of TMD has been associated with a hormonal, physical, and psychosocial change in the adolescent and pubertal stages (4,5). Occlusal conditions have been given less importance, which is not to say that occlusal change does not produce any signs or symptoms of TMD (6). Patients with TMD prior to orthodontic treatment in response to occlusal changes are at increased risk of having signs and symptoms of TMD (7).

Among all dental specialties, orthodontics studies maxillofacial growth and development, dental eruption, and the way these and the maxillary bones relate to each other. There are different reasons for an incorrect bite, such as dental position, jaw bones, and the soft tissues around the anterior teeth, which can also have hereditary components or bad habits. The need for orthodontic treatment in a patient can be determined by the effect that a specific tooth position has on the patient’s health; as well as the effect that the appearance of the teeth has on how they feel about themselves, or both (8,9).

More and more adults are seeking orthodontic treatment, as reported by the British Orthodontic Society, especially females between the ages of 26 and 40 years (10), which are also found in other countries. However, it is also known that there is a greater tendency for females to present TMD compared to males (11). With all of the above, it is important to carry out thorough diagnostic evaluations before orthodontic treatment to determine TMD from the adolescent stage to avoid complications during treatment or any type of medical-legal problem (12).

Some studies have concluded that the occlusal changes produced by orthodontic treatment are not related to the appearance of TMD (13); however, other studies report the finding of signs and symptoms of TMD in patients undergoing orthodontic treatment (14,15). It is important to note that the majority of people receiving orthodontic treatment are children and adolescents, and it is during these developmental stages that TMD is most prevalent. Bearing in mind all of the above, especially about sex and age, it is very difficult to establish a relationship between orthodontics and TMDs; therefore, studies between TMDs and orthodontic treatment should be adjusted to the effect that sex and age may have on patients (2).

The purpose of this systematic review and meta-analysis was to study the association between orthodontic treatment and the occurrence of Temporomandibular Disorders as an update to the previous existing study.

## Material and Methods

- Protocol and registration:

The protocol of the present systematic review was defined a priori by all authors and was developed following the Preferred Reporting Items for Systematic Reviews and Meta-Analyses (PRISMA) guidelines. In addition, the present protocol was registered in the Prospective International Register of Systematic Reviews (PROSPERO) under the registration number CRD42022323328.

To prepare and structure this review, the focus question was developed using the PICO (population, intervention, comparison and outcome) format as detailed below.

• Population: Patients of all ages and both sexes.

• Intervention: Patients with or who had orthodontic treatment.

• Comparison: Patients without orthodontic treatment.

• Outcome: Association between orthodontic treatment and Temporomandibular Disorders.

- Focused question (PICO):

Is there an association between orthodontic treatment and the development of Temporomandibular Disorders?

- Research and selection of studies:

For the present systematic review and meta-analysis, a literature search was performed in five electronic databases Pubmed/Medline, Scopus, Scielo, Google Scholar and Web of Science until April 2022; combining keywords and subject headings according to the thesaurus of each database: “orthodontics”, “orthodontic treatment”, “Temporomandibular Joint Disorders”, “temporomandibular disorders”, “facial pain”, “craniofacial syndromes” (Table 1). In addition, relevant literature was included after a hand search of the references that exist in included studies.

The electronic database search was conducted by two authors (CM, LG) independently, and the final inclusion decision was made according to the following criteria: All case-control and cohort studies, studies dealing with the association between orthodontic treatment and temporomandibular disorders, studies in English and Spanish, studies with no time limit. We excluded articles that were systematic reviews or randomized clinical trials, unpublished studies, and studies reported in more than one publication with different follow-up periods.

**Table 1:**
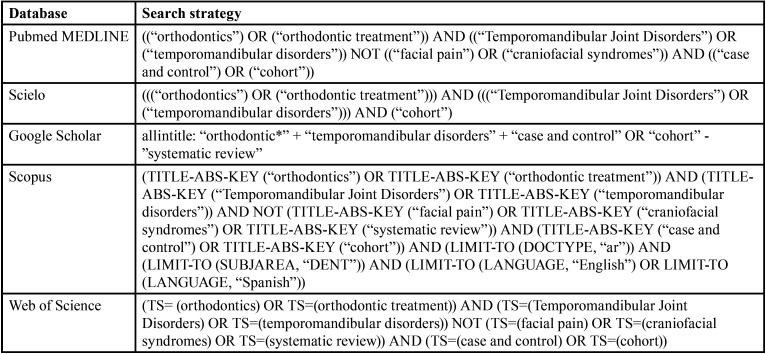
Search strategies for each search engine.

- Data extraction:

A predefined Table was used for data selection for each eligible study, including number, authors, year, study title, number of patients (male/female ratio, mean age (range), follow-up time, groups, number of patients per group, country, results, inclusion criteria and exclusion criteria. From each eligible study, two investigators (LU, JT) independently extracted information and all disagreements were resolved by discussion with a third reviewer (FC).

- Risk of bias assessment:

The risk of bias in the included studies was assessed independently by two calibrated authors (RA, SL) using the NewCastle Ottawa tool adapted for case-control and cohort studies. All disagreements were resolved by discussion with a third reviewer (HA). According to this tool, the domains are assessed on selection, comparability, and exposure/outcomes; and then classified as good quality, fair quality, and poor quality.

- Analysis of results:

Selected study data were entered and analysed in RevMan 5.3 (Cochrane Group, UK), using the ODDS ratio as a measure in a random effects model with a 95% confidence interval. In addition, a GRADE analysis (GRADE Pro GDT, McMaster University and Evidence Prime Inc., Canada) was performed.

## Results

**Figure 1:**
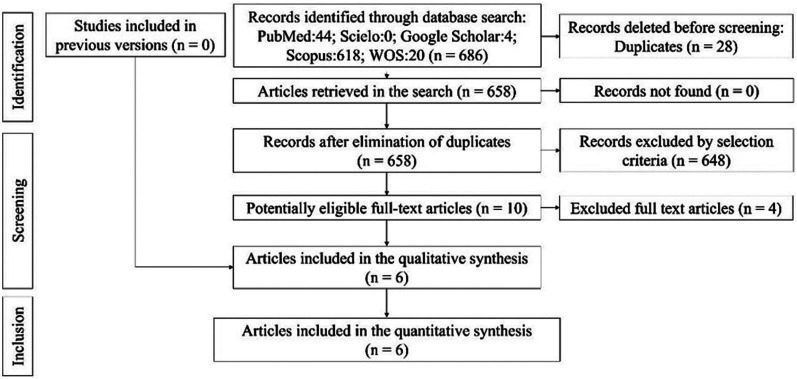
Flowchart.

- Selection of studies:

The electronic and manual search strategy yielded a total of 686 articles, excluding 28 duplicates. After the assessment of the title and abstract, 648 articles were excluded. Ten potentially eligible articles were selected, resulting in the exclusion of four studies, leaving six articles that met the eligibility criteria (case-control and cohorts), which were included for qualitative and quantitative synthesis (Fig. 1). The reasons for study exclusion are listed in Table 2.

**Table 2:**
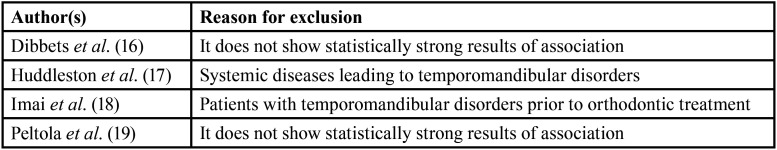
Reason for exclusion of studies.

- Characteristics of the studies included:

Overall, 6 studies (20-25) from 6 different countries were included, of which 4 are case-control, 1 cohort and 1 prospective cohort. One of the most salient features is in relation to the number of patients, with men and women being considered with their respective mean ages and/or according to range. Regarding the extraction of results, all 6 studies (20-25) reported the OR and the confidence interval (CI), while only studies 2 and 3 reported the relative risk, therefore, for the meta-analysis, ORs were considered because all of them had this statistical result (Table 3).

**Table 3:**
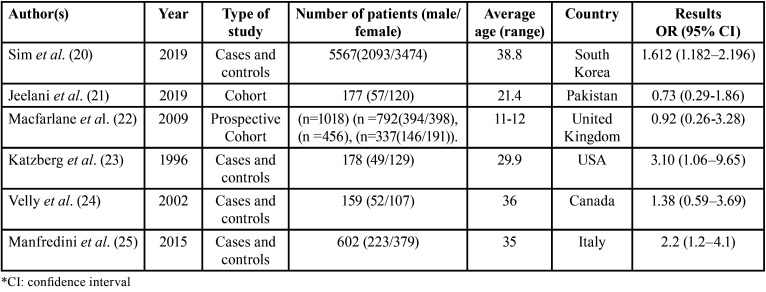
Characteristics of included studies.

- Risk of bias analysis of studies

**Figure 2:**
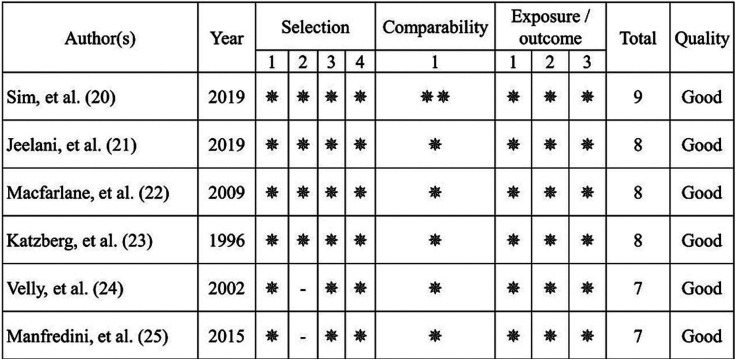
Risk of bias.

In the data extraction process, 6 studies (20-25) were identified as meeting the inclusion criteria, these studies were subjected to risk of bias analysis for case-control/cohort studies, all of which included studies that were of good overall quality according to the domains of selection, compatibility, and outcome and/or exposure (Fig. 2).

- Synthesis of results (Meta-analysis)

**Figure 3:**
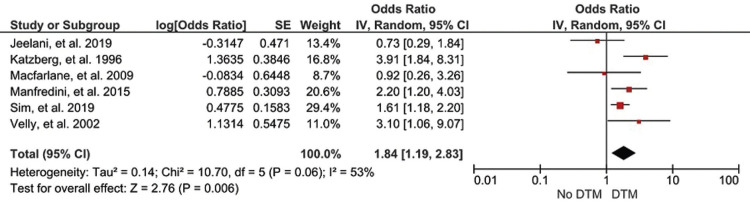
Forest plot.

The association between orthodontic treatment and the occurrence of TMD was determined in 6 studies (20-25), showing that there is a significant association between orthodontic treatment and the occurrence of TMD, equivalent to an association level of 1.84 with a confidence interval of 1.19, 2.83 (Fig. 2). Furthermore, the forest plots show the weights of the 6 studies, where the study by Sim, *et al*. (20) is the one that is giving the most support to the present meta-analysis; however, in most of the studies, there is not distant gap in terms of contribution weights, unlike the study by Macfarlane, *et al*. (22) which only contributed 8.7%. Heterogeneity and the final effect are also observed, where the I2 statistic is equal to 53%; which indicates that, if there is variability due to heterogeneity between the studies, this is corroborated by the final effect which reflects a Z=2.76 with a *p*=0.006; which proves that there is a significant association between orthodontic treatment and the appearance of temporomandibular disorders (Fig. 3).

- GRADE Analysis

When assessing the accuracy of the included studies, it was observed that the GRADE analysis yielded a high overall accuracy, which indicates that the results of the present study are reliable (Table 4).

**Table 4:**
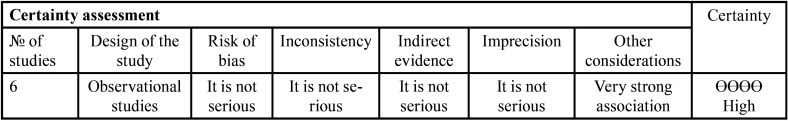
GRADE Analysis.

## Discussion

The aim of this systematic review and meta-analysis was to determine the association between orthodontic treatment and the occurrence of TMD. The results revealed that there is an association between orthodontic treatment and the occurrence of TMD (OR=1.84) and the GRADE analysis showed a high certainty. However, the possible negative effect of orthodontic treatment on the temporomandibular joint has been a matter of concern for orthodontists and dentists in general. At present, the evidence is not clear, and it is mainly accepted that there is no such relationship. Several researchers (40,80) consider that certain dental interventions, including orthodontics itself, may cause TMD. On the other hand, one of the difficulties in diagnosing TMD is the lack of standardization of diagnostic criteria, which makes the comparison between publications difficult (28,29). In contrast to certain findings, the review by Fernández-González *et al*. (28) could not verify the association between a specific type of malocclusion and the occurrence of significant signs and symptoms of TMD and indicated that there seems to be no evidence of a direct or obvious cause-effect relationship between orthodontic treatment and TMD.

Lai, Yap & Türp (29) suggest a high likelihood of orthodontic practitioners encountering individuals with pre-treatment TMD and recommend pre-assessment for TMD before the start of orthodontic treatment. However, Velly, *et al*. (24) using a single protocol in two different clinics and the same evaluator considered bruxism together with grinding, anxiety symptoms, and orthodontic treatment to be factors related to TMD, reporting an OR value of 3.10. In contrast, a cohort study by Jeelani *et al*. (21), reported that there was no significant risk of developing TMD with orthodontic treatment (OR=0.73). However, they emphasized that the occlusal relationships achieved at the end of orthodontic treatment did not remain the same, with a lower degree of relapse.

In a study (22) with 20 years of follow-up, indicate that orthodontic treatment is not related to the onset or prolongation of TMD. Only female sex and the presence of signs and symptoms of TMD during adolescence were the only predictors. The studies identified show a higher prevalence of TMD in females, this is not uncommon as other studies have reported similar findings, so it is presumed that hormonal differences between females and males are responsible, indicating that testosterone may have a protective role in the onset of TMJ pain, unlike estrogen (11,27,28,30). In turn, Sim, *et al*. (20) investigated the relationship between orthodontic treatment and temporomandibular disorders (TMD) in the South Korean population, in which they concluded that undergoing orthodontic treatment is not associated with pain or TMD; however, they found that the orthodontic treatment group showed higher OR values and corresponding 95 % CIs in TMD (OR=1.612). They also indicated that TMD could be related to the age or gender of the patients evaluated and to occlusal interference during orthodontic treatment.

Manfredini *et al*. (25) confirmed the absence of clinically significant effects of orthodontics on TMD (OR=2.2). The finding that orthodontics is not associated with an increased risk of TMD suggests that the concurrence of other factors (31) . On the other hand, Katberg, *et al*. (23), compared the prevalence of internal derangement of the temporomandibular joints (TMJ) in asymptomatic volunteers versus symptomatic subjects using magnetic resonance imaging and found no association between orthodontic treatment and TMD.

It is important to mention that the six articles selected for the present systematic review and meta-analysis had different diagnostic methodologies. In this regard, many different forms of TMD assessment have been proposed in the past, the most commonly used being the Helkimo (32) and currently the Research Diagnostic Criteria for Temporomandibular Disorders (RDC/TMD) (33). The lack of standardization of diagnostic criteria makes the comparison between publications difficult (28,29). However, most research reports a higher prevalence of TMD in people aged over 18 years; because no age group standardization was done in the studies, a maximum age group for TMD in people requiring orthodontic treatment could not be identified, considering that young adults and adolescents constitute the majority of orthodontic patients, there could be a possible age bias (29,30,34-36).

Finally, consideration should be given to certain factors that may increase the painful symptomatology characteristic of TMD in patients with a history of orthodontic treatment. The multifactorial aetiological component of TMD is well known (37), including psychological profile, the age range of patients, gender and hormonal level, habits, and environmental factors (38-40), which need to be evaluated in future work.

## Conclusions

Based on the results of the present systematic review and meta-analysis, it can be concluded that there is an association between orthodontic treatment and the occurrence of TMD; therefore, a person undergoing orthodontic treatment is up to 1.84 times more likely to develop TMD.

## References

[1] (2022). TMD (Temporomandibular Disorders). https://www.nidcr.nih.gov/health-info/tmd.

[2] Rizzante FAP, Duque JA, Duarte MAH, Mondelli RFL, Mendonça G, Ishikiriama SK (2019). Polymerization shrinkage, microhardness and depth of cure of bulk fill resin composites. Dent Mater J.

[3] Yap AUJ, Dworkin SF, Chua EK, List T, Tan KBC, Tan HH (2003). Prevalence of Temporomandibular Disorder Subtypes, Psychologic Distress, and Psychosocial Dysfunction in Asian Patients. J Orofac Pain.

[4] Hirsch C, Hoffmann J, Türp JC (2012). Are temporomandibular disorder symptoms and diagnoses associated with pubertal development in adolescents? An epidemiological study. J Orofac Orthop.

[5] Song YL, Yap AU, Türp JC (2018). Association between temporomandibular disorders and pubertal development: A systematic review. J Oral Rehabil.

[6] Türp JC, Greene CS, Strub JR (2008). Dental occlusion: a critical reflection on past, present and future concepts. J Oral Rehabil.

[7] Bell YL, Jämsä T, Korri S, Niemi PM, Alanen P (2002). Effect of artificial occlusal interferences depends on previous experience of temporomandibular disorders. Acta Odontol Scand.

[8] Batista KB, Thiruvenkatachari B, Harrison JE, O'Brien KD (2018). Orthodontic treatment for prominent upper front teeth (Class II malocclusion) in children and adolescents. Cochrane Oral Health Group, editor. Cochrane Database of Systematic Reviews.

[9] Shaw WC, Richmond S, O'Brien KD, Brook P, Stephens CD (1991). Quality control in orthodontics: indices of treatment need and treatment standards. Br Dent J.

[10] The number of adults seeking orthodontic treatment in the UK continues to rise (2018). Br Dent J.

[11] Bueno CH, Pereira DD, Pattussi MP, Grossi PK, Grossi ML (2018). Gender differences in temporomandibular disorders in adult populational studies: A systematic review and meta-analysis. J Oral Rehabil.

[12] Manfredini D, Bucci MB, Montagna F, Guarda-Nardini L (2011). Temporomandibular disorders assessment: medicolegal considerations in the evidence-based era. J Oral Rehabil.

[13] Ferracane JL (2013). Resin-based composite performance: Are there some things we can't predict?. Dent Mater.

[14] McNamara JA, Seligman DA, Okeson JP (1995). Occlusion, Orthodontic treatment, and temporomandibular disorders: a review. J Orofac Pain.

[15] Hwang SH, Park SG (2018). Experience of Orthodontic Treatment and Symptoms of Temporomandibular Joint in South Korean Adults. Iran J Public Health.

[16] Dibbets JMH, Van Der Weele LT (1989). Prevalence of TMJ symptoms and X-ray findings. Eur J Orthod.

[17] Huddleston Slater JJR, Lobbezoo F, Charlotte Onland-Moret N, Naeije M (2007). Anterior disc displacement with reduction and symptomatic hypermobility in the human temporomandibular joint: Prevalence rates and risk factors in children and teenagers. J Orofac Pain.

[18] Imai T, Okamoto T, Kaneko T, Umeda K, Yamamoto T, Nakamura S (2000). Long-term follow-up of clinical symptoms in TMD patients who underwent occlusal reconstruction by orthodontic treatment. Eur J Orthod.

[19] Peltola JS, Nyström M, Könönen M, Wolf J (1995). Radiographic structural findings in the mandibular condyles of Young individuals receiving orthodontic treatment. Acta Odontol Scand.

[20] Sim HY, Kim HS, Jung DU, Lee H, Han YS, Han K (2019). Investigation of the association between orthodontic treatment and temporomandibular joint pain and dysfunction in the South Korean population. Korean J Orthod.

[21] Jeelani W, Habib A, Ali B, Ahmed M (2019). Effect of Orthodontic Treatment With Straight-Wire Appliance on the Incidence of Temporomandibular Disorders-A 3-Year Cohort Study. J Indian Orthod Soc.

[22] Macfarlane TV, Kenealy P, Kingdon HA, Mohlin BO, Pilley JR, Richmond S (2009). Twenty-year cohort study of health gain from orthodontic treatment: temporomandibular disorders. Am J Orthod Dentofacial Orthop.

[23] Katzberg RW, Westesson PL, Tallents RH, Drake CM (1996). Orthodontics and temporomandibular joint internal derangement. Am J Orthod Dentofacial Orthop.

[24] Velly AM, Gornitsky M, Philippe P (2002). A case-control study of temporomandibular disorders: symptomatic disc displacement. J Oral Rehabil.

[25] Manfredini D, Stellini E, Gracco A, Lombardo L, Nardini LG, Siciliani G (2016). Orthodontics is temporomandibular disorder-neutral. Angle Orthod.

[26] Godoy F, Rosenblatt A, Godoy-Bezerra J (2007). Temporomandibular disorders and associated factors in Brazilian teenagers: A cross-sectional study. Int J Prosthodont.

[27] Luther F (1998). Orthodontics and the temporomandibular joint: where are we now? Part 2. Functional occlusion, malocclusion, and TMD. Angle Orthod.

[28] Fernandez-Gonzalez F, Canigral A, Lopez-Caballo J, Brizuela A, Moreno-Hay I, del Rio-Highsmith J (2015). Influence of orthodontic treatment on temporomandibular disorders. A systematic review. J Clin Exp Dent.

[29] Lai YC, Yap AU, Türp JC (2020). Prevalence of temporomandibular disorders in patients seeking orthodontic treatment: A systematic review. J Oral Rehabil.

[30] LeResche L, Saunders K, Von Korff MR, Barlow W, Dworkin SF (1997). Use of exogenous hormones and risk of temporomandibular disorder pain. Pain.

[31] Mušanović A, Ajanović M, Redžepagić Vražalica L, Kazazić L, Tosum Pošković S, Mlačo Durek J (2021). Prevalence of TMD among Children Provided with Fixed Orthodontic Treatment. Acta Stomatol Croat.

[32] Helkimo M (1974). Studies on function and dysfunction of the masticatory system. II. Index for anamnestic and clinical dysfunction and occlusal state. Sven Tandlak Tidskr.

[33] Dworkin SF, LeResche L (1992). Research diagnostic criteria for temporomandibular disorders: review, criteria, examinations and specifications, critique. J Craniomandib Disord.

[34] Egermark I, Carlsson GE, Magnusson T (2005). A prospective long-term study of signs and symptoms of temporomandibular disorders in patients who received orthodontic treatment in childhood. Angle Orthod.

[35] LeResche L, Sherman JJ, Huggins K, Saunders K, Mancl LA, Lentz G (2005). Musculoskeletal orofacial pain and other signs and symptoms of temporomandibular disorders during pregnancy: a prospective study. J Orofac Pain.

[36] Fischer L, Clemente JT, Tambeli CH (2007). The protective role of testosterone in the development of temporomandibular joint pain. J Pain.

[37] Slade GD, Bair E, Greenspan JD, Dubner R, Fillingim RB, Diatchenko L (2013). Signs and Symptoms of First-Onset TMD and Sociodemographic Predictors of Its Development: The OPPERA Prospective Cohort Study. J Pain.

[38] Maixner W, Diatchenko L, Dubner R, Fillingim RB, Greenspan JD, Knott C (2011). Orofacial Pain Prospective Evaluation and Risk Assessment Study - The OPPERA Study. J Pain.

[39] Bair E, Gaynor S, Slade GD, Ohrbach R, Fillingim RB, Greenspan JD (2016). Identification of clusters of individuals relevant to temporomandibular disorders and other chronic pain conditions: the OPPERA study. Pain.

[40] Meloto CB, Slade GD, Lichtenwalter RN, Bair E, Rathnayaka N, Diatchenko L (2019). Clinical predictors of persistent temporomandibular disorder in people with first-onset temporomandibular disorder: A prospective case-control study. J Am Dent Assoc.

